# Fifteen years and counting: looking towards the future

**DOI:** 10.1186/s13244-024-01873-8

**Published:** 2025-01-01

**Authors:** Paola Clauser

**Affiliations:** Medical University of Vienna, Vienna, Austria

Fifteen years ago, Prof. Robert Hermans and Prof. Adrian K. Dixon wrote an enthusiastic editorial, welcoming the second journal of the ESR Journal Family, *Insights into Imaging* [1]. Prof. Hermans served as the first Editor-in-Chief of the journal, which was introduced to create space for educational articles, such as pictorial reviews and society policy statements, that often did not find sufficient space or visibility in *European Radiology*. Since then, *Insights into Imaging*, alongside *European Radiology* and the newest ESR journal, *European Radiology Experimental*, has grown to become one of the top journals in the medical imaging field.

An essential factor to this success has been the energetic and visionary guidance of Prof. Luis Martí-Bonmatí, who took over from Prof. Hermans in January 2018. After his appointment, Prof. Martí-Bonmatí expanded the focus of the journal, placing particular attention on critical thinking [2].

As highlighted in a recent editorial [3], the practice of medicine needs to be based not only on ample knowledge of the current evidence, recommendations, and guidelines but also on clinical experience. It is only when we deeply reflect on our practice, combining current knowledge and our clinical experience, that we can identify the relevant gaps, move the science forward, and improve our practice. Only by critically revising our work by constantly questioning previous knowledge, we can improve [3].

An opinion recently published in the BMJ [4] stated something of common sense: medicine is difficult. Diagnosis is a challenging process that cannot only be based on image description and established guidelines. Relevant clinical information and contexts are fundamental for reaching the correct diagnosis. The role of radiologists is central to making diagnoses and guiding therapeutic decisions. Radiologists need to learn to work in a multidisciplinary team and to be aware of all the steps of the diagnosis and treatment to provide a significant contribution to patients’ management.

As I make my first steps as the newly appointed Editor-in-Chief of *Insights into Imaging*, guided by the invaluable support of my predecessor, this editorial is the perfect opportunity to once again emphasize the role of *Insights into Imaging* as a source for all radiologists, irrespective of the work setting, to find precise information on cutting edge radiology and reflection on how to improve our current practice.

In the last few years, the concept of evidence-based medicine has gradually evolved into the concept of value-based medicine. Practicing value-based radiology means practicing radiology while considering the evidence but also the benefits conferred on patients and society [5]. I believe this concept is an essential part of the aims and scope of the journal moving forward. Medicine, in general, but radiology, in particular, is rapidly evolving. Radiology, being based on technology, is probably the field in which fast changes, with artificial intelligence introduction and fast and continuous development of new radiological devices, are more apparent. These changes could be a beneficial support at a time when the radiologist shortage is becoming apparent in many European countries, particularly in the academic setting [6]. The only way in which we can successfully face these challenges is by moving out of our reporting rooms. We are not doctors’ doctors, only seeing society through our (admittedly, now often rather colorful) screens [7]. We are clinicians who play a central role in patient management and decision-making, and it is our responsibility to behave as such and train the next generation for this role.

As the Editor-in-Chief, together with a growing and passionate Editorial Team, we will keep the focus of the journal on high-quality review and educational articles, to refresh basic knowledge but also to facilitate the translation of new methods and technologies into clinical practice [8].

Open access publishing can be onerous for authors, but it allows the published work to reach a wider audience that goes beyond academic institutions. It is in the interest of our journal to provide the readers with educational and critical reviews from experts in the field, and the ESR will continue to support its members with funding options in this endeavor.

The journal will also increase its focus on multidisciplinarity, supporting radiologists in keeping up-to-date with the upcoming novelties outside the field of radiology, complementary to the “ESR Bridges” series from *European Radiology* [9]. In addition, *Insights into Imaging* will further increase its focus on educational, review, and original articles on resource optimization, equity of access to care, and sustainability.

I am eager to work with the *Insights into Imaging* team to continue providing the best knowledge on current radiology and supporting critical thinking and value-based practice. We hope that we will continue to have the support of the authors and reviewers who, with their hard work, provide a vital contribution to the success of this journal. With their continuous and invaluable support, we will be able to reach an even wider audience, increasing awareness of and providing guidance on the role of radiology in the multidisciplinary team and on the current challenges of radiology and medicine in our society.

## Data Availability

Not applicable.

## References

[1] Hermans R, Dixon AK (2010) Editorial: a new journal in radiology. Insights Imaging 1:1. 10.1007/s13244-009-0004-0 22347896 10.1007/s13244-009-0004-0 PMC3259413

[2] Martí-Bonmatí L (2018) Letter from the new editor-in-chief for Insights into Imaging. Insights Imaging 9:119–120. 10.1007/s13244-018-0617-2 29594849 10.1007/s13244-018-0617-2 PMC5893495

[3] Martí-Bonmatí L (2023) Embracing critical thinking to enhance our practice. Insights Imaging 14:97. 10.1186/s13244-023-01435-4 37222825 10.1186/s13244-023-01435-4 PMC10209363

[4] Elder A, Royal College of Physicians of Edinburgh (2024) Medicine is difficult—there are no shortcuts. BMJ 387:q2163. 10.1136/bmj.q2163 39362674 10.1136/bmj.q2163

[5] European Society of Radiology (ESR) (2021) Value-based radiology: what is the ESR doing, and what should we do in the future? Insights Imaging 12:108. 10.1186/s13244-021-01056-9 34318401 10.1186/s13244-021-01056-9 PMC8316510

[6] Zhong J, Ho R, Gourtsoyianni S et al (2022) Attracting the next generation of radiologists: a statement by the European Society of Radiology (ESR). Insights Imaging 13:84. 10.1186/s13244-022-01221-8 35507198 10.1186/s13244-022-01221-8 PMC9066129

[7] Glazer GM, Ruiz-Wibbelsmann JA (2011) The invisible radiologist. Radiology 258:18–22. 10.1148/radiol.10101447 21183490 10.1148/radiol.10101447

[8] Smits M, Rockall A, Constantinescu SN et al (2024) Translating radiological research into practice-from discovery to clinical impact. Insights Imaging 15:13. 10.1186/s13244-023-01596-2 38228934 10.1186/s13244-023-01596-2 PMC10792138

[9] Hamm B (2024) Navigating challenges and opportunities: a new era for European Radiology. Eur Radiol 34:3–5. 10.1007/s00330-023-10486-6 38165431 10.1007/s00330-023-10486-6

